# Preliminary studies on high potential narrow-bandgap *Parkia biglobosa* pod husk natural dye extracts for high-performance DSSCs

**DOI:** 10.1039/d5ra03406j

**Published:** 2025-07-16

**Authors:** Pascal Nbelayim, Go Kawamura, Tan Wai Kian, Grace Ngubeni, George Hasegawa, Boateng Onwona-Agyeman, Kazuki Nakanishi, Nosipho Moloto, Pierre Kalenga Mubiayi, Atsunori Matsuda

**Affiliations:** a Department of Electrical and Electronic Information Engineering, Toyohashi University of Technology 1-1 Hibarigaoka, Tempaku-cho Toyohashi Aichi 441-8580 Japan psfuseininbelayim@ug.edu.gh +81-532-44-6800; b Institute of Materials and Systems for Sustainability, Nagoya University Furo-cho, Chikusa-ku Nagoya Aichi 464-8601 Japan +81-527893920; c Department of Materials Science & Engineering, School of Engineering Sciences, University of Ghana Legon Accra 00233 Ghana +233 302213820/50 +233271000577; d School of Chemistry, Faculty of Science, University of Witwatersrand 1 Jorissen St. Johannesburg South Africa +27 11 717 1888

## Abstract

The urgent global transition away from fossil fuels, driven by climate change mitigation, rising energy demands, and exponential growth of high-energy-consuming AI technology, calls for every available sustainable renewable energy solution. DSSCs emerge as promising photovoltaics due to their cost-effectiveness, efficiency in low-light conditions, versatility, and aesthetic appeal. A crucial avenue for enhancing DSSC sustainability lies in utilizing natural dyes as sensitizers. This study explores novel natural dye extracts from the pod husk of *Parkia biglobosa*, employing seven different solvents to investigate their photovoltaic potential. Some extracts exhibit exceptional light absorbance with band gaps ranging 1.82–2.85 eV—comparable to the high-performing synthetic N719 dye (1.75 eV). These performances surpass typical natural dyes with band gaps ≥2.0 eV. Photovoltaic performance assessments yielded efficiencies between 0.07–0.19%, within the reported range of natural dye DSSCs (0.05–4.2%; usually of high purity or combinations), though significantly lower than N719 (6.22%). Photoanode thickness reduction from 8 to 5 μm enhanced efficiencies to 0.09–0.24% (compared to 4.20% for N719), yet fell below anticipated values based on strong optical absorption. Subsequent characterizations—TG-DTA, IPCE, PL, EIS, FT-IR, and CV—identified two primary limiting factors: high series and ion diffusion resistances, attributed to inefficient band alignments with TiO_2_ and the I_3_^−^/I^−^ electrolyte, and dye degradation. Optimizing DSSC architecture through appropriate semiconductor materials and redox electrolytes could significantly improve these natural dyes performances. This work advances the potential for cost-effective, eco-friendly, high-performance DSSCs and contributes to groundwork for future advancements in sustainable solar energy.

## Abstract 2

The urgent global transition away from fossil fuels, driven by climate change mitigation, rising energy demands, and exponential growth of high-energy-consuming AI technology, calls for every available sustainable renewable energy solution.

## Graphical abstract

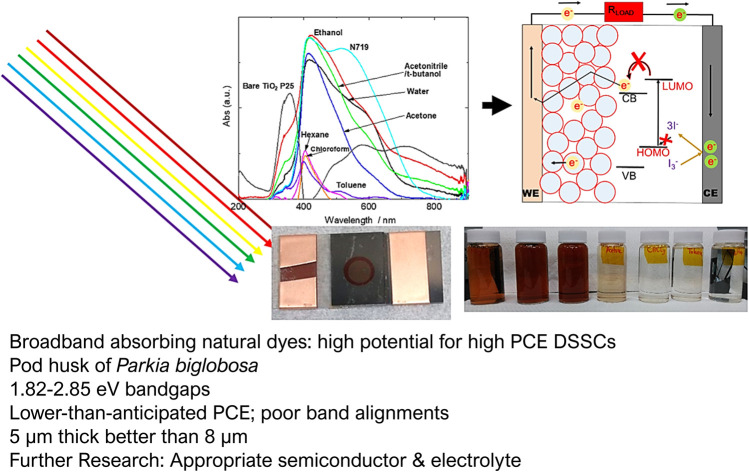

## Introduction

The pursuit of sustainable renewable energy sources has intensified as the global reliance on fossil fuels (which currently accounts for 81.5% (ref. 1) of total energy consumption) becomes increasingly untenable due to their severe adverse environmental impacts, non-renewable nature, and rapid depletion due to growing economies and populations. According to the International Energy Agency's (IEA) 2024 Electricity Mid-Year Update report,^2,3^ electricity demand is projected to increase by 4% in 2024 and 2025, fueled by robust economic growth, intense heatwaves, and ongoing electrification worldwide. Consequently, there is an urgent and critical need for sustainable renewable energy sources to address the energy transition and meet the demands of global population growth and rapidly emerging economies.

Common available techniques for renewable energy include thermal, biomass, mechanical, solar and electrochemical. The solar/photovoltaics (PVs) have the unique advantage of a free abundant principal source (the sun) of 3.89 × 10^26^ J s^−1^ (with 1.367 kW m^−2^ reaching the earth). Dye-sensitized solar cell (DSSC) technology is among the sub-set of the 3^rd^ generation emerging PV technologies^4^ and an established viable alternative solar cell technology; from the unprecedented power conversion efficiency (PCE) of 7.1% in 1991 by O'Regan & Grätzel.^5^ This has gradually improved over the years with a current certified record PCE of 11.9% by SHARP^6^ and an uncertified record of ∼14.3%.^7,8^

The DSSC was presented as an alternative to the traditional solid state, mainly silicon-based, solar cells with the unique characteristics of low-cost and environmental friendliness. However, the cost of Si production has reduced, by at least 90% since 2008,^9^ for the Si-based 1^st^ generation PVs (which constitute the main PV market of about 97% (ref. 10)). Nevertheless, DSSC still remains a facile and low-cost fabrication technology. In addition, with regards to ambient and indoor light conditions, esthetic use, environmental friendliness and Building-Integrated PVs (BIPVs), DSSC outperforms all other solar cell technologies,^11,12^ although perovskite solar cells (PSCs) technology seriously competes in these core key advantages of DSSC over other solar cell technologies, and with impressive efficiencies, over 26%,^13^ However, PSCs has more severe stability challenges, although being gradually improved.^14^

A core component of the DSSC is a light absorbing dye/sensitizer anchored on a wide bandgap mesoporous semiconductor material (SCM; typically, TiO_2_) by an anchoring ligand of the dye. The function of the dye is to absorb photons to generate electron–hole pairs, and the electrons injected into the conduction band (CB) of the SCM *via* the anchoring ligand. These electrons are then transported to the external circuit of the solar cell, and by that generate electricity. This sets the dye as a principal component of the DSSC. An efficient dye should have:^15^

1. Good absorption coefficient to absorb light across the UV-NIR (300–930 nm) region.

2. An effective anchoring group to bind and transfer electrons to the SCM.

3. A fast-rate excited-electron transfer to the SCM than its decay-rate.

4. Appropriate energy band edges alignment of its LUMO to the conduction band (CB) of the SCM, and HOMO to the reduction potential of the electrolyte, for efficient electron and hole transfers, respectively.

**Fig. 1:**
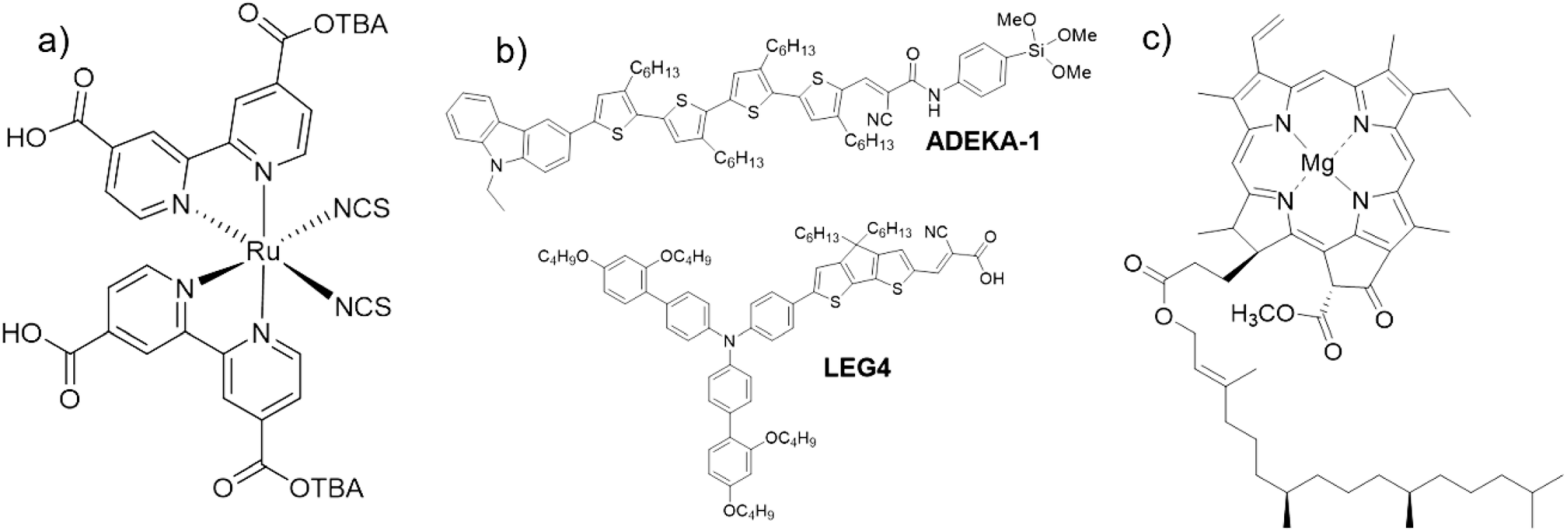
Examples of chemical structures of the three main categories of dyes used in DSSC: (a) metal complex dyes (N719 dye); (b) metal-free organic dyes (ADEKA-1 and LEG4 (ref. 7)); (c) natural dyes (chlorophyll a).

Currently, there are generally three categories of dyes employed in DSSCs: synthetic metal-complex dyes, synthetic metal-free organic dyes and natural dyes^11,19^ (Fig. 1a–c). The metal complex dyes (*e.g.* N719 (di-tetrabutylammonium *cis*-bis(isothiocyanato) bis(2,2′-bipyridyl-4,4′-dicarboxylato)ruthenium(ii) in Fig. 1a), N3, and N749 dyes) are the best performing dyes, but involve complex and inefficient syntheses, expensive catalysts, and expensive rare/noble earth metals.^11,19^ Thus, they are relatively uneconomical and unsustainable. The metal-free organic dyes received attention around the year 2000 when they increased PCEs from 4 to 9%,^19^ and now they have the record DSSC PCE of 14.3%,^7,8^*via* a combination of different kinds of this group of dyes such as ADEKA-1 and LEG4 (Fig. 1c) in the works of Kakiage *et al.*^7^ However, their syntheses are tedious, and their products and by-products are potential environmental pollutants.^11^ They can be unstable and can also be considered unsustainable. The natural dyes are obtained from nature (usually from various parts of plants in the form of chlorophylls (*e.g.* chlorophyll a, Fig. 1b), betalains, flavonoids, carotenoids, *etc.*). They are easily and highly available, involve facile and low-cost preparation techniques, are environmentally friendly, and are biodegradable.^11,19^ However, they have low efficiencies (0.04–4.2% (ref. 19)) with stability issues.

**Fig. 2:**
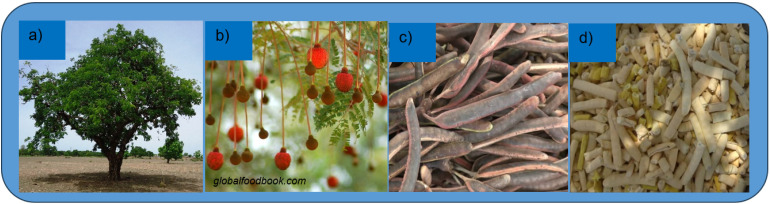
Parts of *Parkia biglobosa* plant: (a) the plant; (b) the flower; (c) the fruit with the pod husk cover; (d) the pulp after removing the pod husk.

This work uses natural dye extracts for DSSCs from the pod husk of a plant, *Parkia biglobosa* (PB), shown in Fig. 2a; some of which extracts have broadband light absorbance across 700 to 400 nm range of the solar spectrum. This is unusual for natural dyes^11,19^ as most of the reported natural dyes usually have a total of just about 300 nm ranges absorbance within the solar spectrum.

Chang *et al.*^16^ reported on natural dyes extracts DSSCs of chlorophyll and anthocyanin from pomegranate leaf and mulberry fruit, respectively, *via* warm ethanol soaking extraction. The extracted chlorophyll dye exhibited major light absorbance in the regions of 700–600 nm and 500–400 nm of the solar spectrum, to give a PCE of 0.6%, while the anthocyanin dye exhibited broadband light absorbance similar to the popular N719 dye, but with low power conversion within the solar spectrum region range of 550–450 nm to give a PCE of 0.5%. A cocktail mixture of the two natural dyes seems to equal the absorbance of N719 except in the range of 470–550 nm, giving a PCE of 0.7%. The works of R. Syafinar *et al.*,^17^ showed that chlorophyll extracted from spinach with ethanol had better light absorbance with band gap of 1.83 eV than with water extraction of band gap of 1.88 eV. X.-F. Wang *et al.*^18^ used the effect of cascading charge transfer by use of carotenoids as additional redox component/spacers to add additional redox component between a natural dye sensitizer and the I^−^/I_3_^−^ redox couple. The carotenoids were deposited on a TiO_2_ photoanode in combination with a chlorophyll-a derivative sensitizer (methyl 3-devinyl-3-carboxy-pyropheophorbide a). These carotenoids were observed to enhance the PCE from below 3.6% of no-carotenoid sample to a maximum of 4.2% of β-carotene-spiked photoanode sample, with no evidence of energy transfer from β-carotene to the sensitizer. The carotenoids were also suggested to play an additional photo-protective role of suppressing or eliminating the formation of radicals. The derived natural dye sensitizer had most of its light absorbance in the regions of 700–750 nm and 500–350 nm within the solar irradiance spectrum.

In this work, we made eight natural dye extracts from the pod husk of PB, using seven different polar and non-polar solvents, which exhibited broadband light absorbances across 700–300 nm range of the solar spectrum. Some of these extracts displayed almost the same spectrum as the high-performing metal-complex synthetic N719 dye. Thus, we hypothesized high-performance DSSCs from these dyes comparable to N719 dye.

The PB plant (Fig. 2a) has a wide distribution range across the Sudan and Guinea savanna ecological zones.^20^ It has red-brown flowers (Fig. 2b) and red-to-brown pod husks (Fig. 2c).

The various parts of PB are mainly investigated for their nutritional and phytochemical uses which include: the leaves, used as fodder; the fresh twigs for oral hygiene/cleansing, and treatment of tooth- and ear-aches; the wood for furniture and biofuel; the pod husk (Fig. 2c) extracts for esthetic, binding and hydrophobic effect treatments on buildings, and as a pesticide; the pulp (Fig. 2d), contains 60% sugar, is used as porridge or sweetener; the seed (located inside the pulp, Fig. 1d), is used for the preparation of condiment or tea.^21–23^ Other publications on this versatile plant include ref. 24–26.

## Experimental

### Dye extraction

**Fig. 3:**
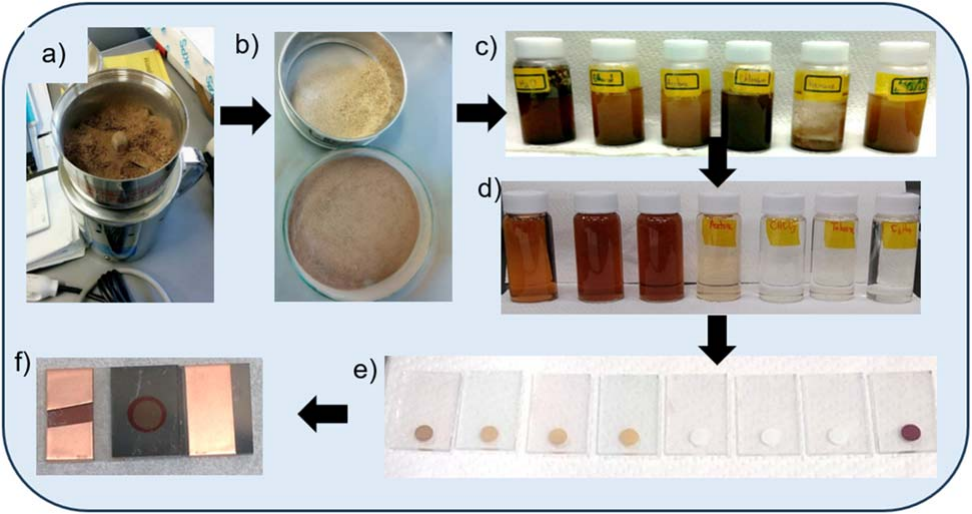
A flow diagram showing the experimental processes (from top left): (a) milling/pulverizing, (b) sieving, (c) soaking/extraction, (d) after centrifuging, (e) dyeing of TiO_2_ coatings into photoanodes, including N719 dye (first from right), and (f) an assembled DSSC.

Sun-dried PB pod husk was harvested (Northern region, Ghana), cut into pieces and pulverized (Wonder blender; DM-6) for about 2 min (Fig. 3). The obtained powder was sieved (106 μm aperture), dried at 60 °C for 24 h in a convection oven and stored in an air-tight glass vial in the dark.

To each of seven 50 ml-capacity vials was added 0.5 g of the powder and 20 ml aliquots of the respective solvents (Fig. 3): water; ethanol; 1 : 1 acetonitrile/*t*-butanol; acetone; chloroform; toluene; and hexane [all Wako; reagent grades]. The solvent-powder mixture suspensions were shaken vigorously and sonicated for 30 min, sealed and stored in a dark cupboard for 1 week to extract the dyes.

The mixtures were then centrifuged [Kubota 7000] at 10 000 rpm for 5 min in polypropylene and Teflon vials. The supernatant solvents were decanted into volumetric flasks and made up to 100 ml with their respective extraction solvents as the final dye solutions. These were stored in a refrigerator when not in use. A 0.3 mol L^−1^ N719 dye [ALDRICH; 65 mol% dioxole] was prepared in ethanol as a reference dye.

### Pastes and photoanodes preparation

The paste was prepared by adding 1 g of TiO_2_ P25 Degussa [Sigma-Aldrich; 21 nm; ≥99.5%] to 1.74 ml of ethylene glycol (dehydrated, Wako; 99.5% min) and milled using a planetary ball mill. After 1 h, 1.69 g citric acid (Chameleon reagent; anhydrous, ≥99.5%) was added and continued milling to form the coating paste. For the photoanode preparation, fluorine-doped tin oxide (FTO)-coated glass substrates [85% T; 9 Ω □^−1^; 3 × 2 cm] were first cleaned by RCA treatment and coated with a TiO_2_ buffer layers, by treating them in 0.04 mol L^−1^ TiCl_4_ (Wako; 99.0% min) at 70 °C for 30 min. These coated substrates were washed with water, dried and heat-treated at 450 °C for 1 h. The paste was then coated onto these treated FTO substrates by doctor blade coating with circular active areas of 0.282 cm^2^ (*ø* = 6 mm), according to our previous work.^25^ For uniformity and reproducibility, these coatings were done using a mechanical drawing of the doctor blade at a rate of 2.5 mm s^−1^ to spread the paste. The thickness of the photoanode was controlled by loading specific weights on the doctor blade holder. The coated samples were dried on a hot plate, sequentially: at room temperature, 60 °C & 120 °C, for 5 minutes each. These were then calcined at 500 °C for 1 h, washed with ethanol and dried with a hot blower. For dye sensitization, six (6) samples each of the dried samples were immersed in the various dye extracts from Fig. 3, including the N719 dye, for 24 h. The dye-sensitized photoanodes were rinsed with their individual pristine extracting solvents to remove excess dye, and dried using a hot blower.

### DSSCs assemble

Counter electrodes of Pt on RCA-treated FTO (with two pinholes of 2 mm apart for electrolyte filling) were prepared by sputter coating at 15 mA for 600 s. Sandwich-type cells were prepared using a 50 μm plastic spacers (DuPont, Himilan), with open circular areas of 0.5 cm^2^ (*ø* = 8 mm), at 105 °C.

The cell fabrication was completed by injecting I^−^/I_3_^−^ electrolyte prepared in acetonitrile solvent and composed of: 0.05 mol L^−1^ iodine (Aldrich; ≥99%), 0.1 mol L^−1^ lithium iodide (Strem Chemicals; anhydrous, 98% min), 0.6 mol L^−1^ 1, 2-dimethyl-3-propylimidazolium iodide (TCI; >98%), and 0.5 mol L^−1^ 4-*tert*-butylpyridine (Aldrich; 96%).The cells were then sealed with a piece of the spacer and scotch tape, similar to our previous work.^31^

### Characterization

The light absorbance characteristics of the dyes were measured using UV-vis spectroscopy (JASCO V-600 *via* both transmission and diffuse reflectance spectroscopy (DRS) modes). Their bandgaps were estimated using the Kubelka–Munk theory using data from the DRS UV-vis absorbance measurements of their photoanodes.

Photoluminescence (PL) spectroscopy characterization was carried out using a He–Cd laser (325 nm) as the excitation source. The luminescence was processed through a single monochromator grating to a multi-channel charge-coupled device (CCD)-array detector at −60 °C using “Andor Solis 32 bit” software and “Stream Basic” software-operated microscope.

A thermogravimetric-differential thermal analysis (TG-DTA) equipment [Rigaku Thermo plus EVO2; TG 8120] was used to study the thermal characteristics of the pod husk/dye and N719 dye powders.

The current–voltage (*I*–*V*) characteristics of the cells were evaluated using ADCMT 6244 DC Voltage/Current Source/Monitor and an HAL-320 W solar simulator (Asahi spectra) with an air-mass 1.5 global filter. The solar simulator was calibrated to an intensity of 100 mW cm^−2^ (1 sun), using a 1 SUN checker (Asahi CS-40). Five (5) samples for each dye extract sample were measured and the average recorded; for each batch and modification (pH, photoanode thickness, *etc.*). A masking with a circular open active area of 0.282 cm^2^ was used.

The Incident Photon to Current Conversion Efficiency (IPCE) of the solar cells was evaluated using Bunkoukeiki SM-250KB spectrometer with a Keithley 2401 source meter using an irradiation flux of 2.0 × 10^15^ photons, with a masking of circular open active area of 0.282 cm^2^. The average of five sample readings per dye extract measurement was recorded.

An attenuated total reflection (ATR) mode [Shimadzu GATR 10] Fourier Transform Infrared Spectroscopy (FT-IR) spectrometer [IR Tracer-100, Shimadzu Co., Japan] was used to evaluate for the functional group composition of the dyes.

The electron charge dynamics of the solar cells was evaluated by electrochemical impedance spectroscopy (EIS) under 1 sun at open circuit voltage (OCV) bias within a frequency range of 0.1 Hz to 1 MHz, and an AC amplitude of 40 mV (obtained by iteration).

Scanning electron microscopy (SEM) [JEOL JSM-6060S, JEOL Ltd] was used to determine the thickness of the TiO_2_ photoanodes from cross-sectional cuttings of these photoanodes.

The inorganic elemental composition of ash residues of the dyes was evaluated *via* energy dispersive spectroscopy (EDS) detector [JED-2300, JEOL Ltd] attached to the SEM equipment.

The cyclic voltammetry evaluation of the dyes were measured in acetonitrile with 0.15 mol L^−1^ tetrabutylammonium hexafluorophosphate (Bu_4_NPF_6_) as electrolyte (working electrode: TiO_2_ P25/dye on glassy carbon, 3 mm; reference electrode: ALS RE-7 Ag/Ag^+^ non-aqueous; calibrated with ferrocene/ferrocenium (Fc/Fc^+^) and as an external reference; counter electrode: Pt) at 50 mV s^−1^, for at least 3 cycles each; using HD Hokuto Denko HSV automated polarization system.

## Results and discussion

### Thermogravimetry

**Fig. 4:**
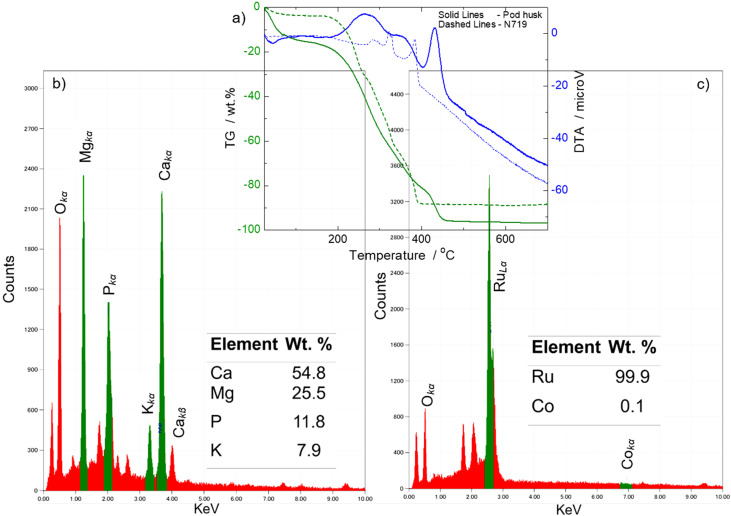
(a) (overlay on (b) and (c)) TG-DTA (1 °C min^−1^) curves of the pod husk powder showing it's thermal stability, comparable to that of 719 dye powder; (b) and (c) SEM-EDS spectra and tabulated quantitative values (inserts) of residue powders from the TG-DTA characterizations showing their inorganic elemental compositions.

We studied thermal characteristics (Fig. 4a) of the pod husk powder, to evaluate its comparative thermal stabilities and organic/inorganic compositions with that of N719 dye powder. The pod husk composite of the natural dyes (pod husk powder) showed initial weight loss of about 13% (from an initial mass of 9.8 mg), from room temperature to 80 °C, with an accompanying endothermic peak. This is attributed to loss of volatile and moisture components. This significant loss was not observed in the N719 dye; only about 2.3 wt% loss (from an initial mass of 2.8 mg) up to 56 °C. This is ascribed to its analytical grade and non-hygroscopic nature, such that it does not large amount of absorbed/adsorbed moisture. However, between 186–400 °C, the N719 dye loses about 85.7 wt% with three exothermic peaks indicating the stepwise combustion loss of functional groups of thiocyanide, carboxylic and aromatic pyridyl ligands,^32–35^ respectively. This leaves an ash content of about 12 wt%, which we attribute to the inorganic Ru metal complexing component. Theoretically, it should be about 8.5 wt%. Thus, the 12 wt% suggests it has been converted to its oxide form, given the air–atmosphere thermogravimetric treatment. The pod husk results also show three exothermic peaks with accompanying loss of 83 wt% between 186–450 °C. This mass loss is attributed to combusted organic component of the dye powder. However, since the chemical compounds/composition of the powder (dye components) have not been fully determined yet, we are unable to assign these exothermic peaks to particular functional groups. However, the works of Abagale S. A. *et al.*^22^ and Fayinminnu O. O. *et al.*,^23^ with phytochemical-interest bias, identified families of compounds such as flavonoids, saponins and tannins as main components of the polar extracts, with suspected presence of low molecular weight carboxylic acids, alkenes, alkynes, alcohols, ketones, aldehydes, nitro compounds, esters, ethers, amides. The results also show an inorganic ash content of about 4 wt%; indicating a highly organic dye material.

### SEM-EDS

The ash content of the powders after 1000 °C were analyzed for their elemental composition *via* SEM-EDS. The results are shown in Fig. 4b and c. It was found that the ash content of the N719 dye was basically Ru element (99.9%, inset table of Fig. 4c as expected. The EDS spectrum of Fig. 4c also shows a peak around 0.52 eV, corresponding to oxygen (which corroborates our assertion of the formation of Ru oxides); and another peak around 0.28 eV, which we attribute to a false carbon content from the material component of the EDS window instrumentation or atmospheric carbon materials during SEM sample preparation of the ash. The ash content of the natural dyes composite showed elemental composition of Ca, Mg, P and K (inset of Fig. 4b), with Ca and Mg as the main components. This result is typical of the inorganic composition of plant materials.^29–32^ Its EDS spectrum also showed peaks of oxygen that suggest the elements are in their oxide forms, probably from the air–atmosphere ashing/gravimetry. These results also suggest that these natural dyes could be of metal–organic complex forms such as N719 dye or chlorophyll (Fig. 1), or simply as dye impurity species.

These results show comparable thermal characteristics of the natural dyes to the standard N719 dye, and indicate that these dyes are thermally stable, at least, up to 186 °C, and thus, will survive the highest heat-treatment temperature step of 120 °C used in the DSSC assemble.

### Optical studies

**Fig. 5:**
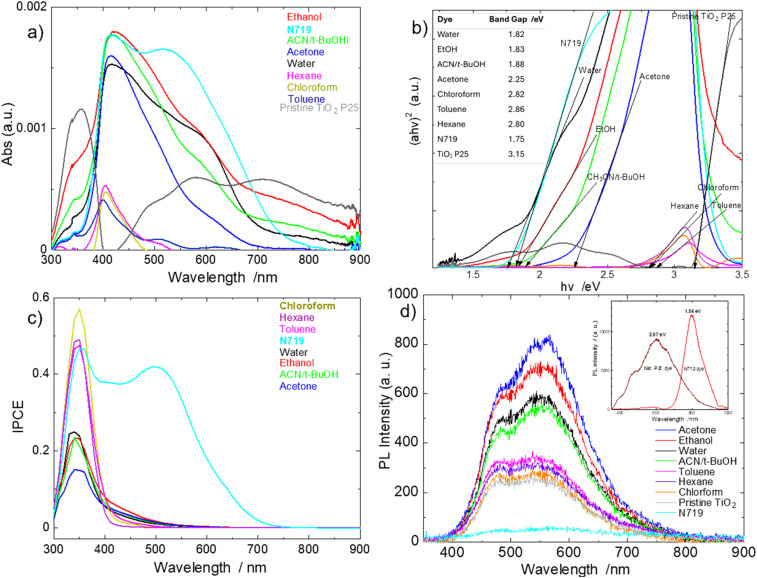
(a) Broadband Light absorbance spectra of dye extracts from the photoanodes with some of the natural dyes showing competitive broadband absorbance to N719 dye; (b) Tauc plot-estimated bandgaps of pristine dyes from dyed photoanodes also showing close optical performance potential to N719 dye; inset: quantitative band gap values table; (c) IPCE of DSSCs of extracted dyes showing unexpected relatively lower performance to N719 dye in the visible region; (d) photoluminescence spectra of dyed photoanodes showing natural dye extracts with high photogenerated electrons but with high recombination; inset: direct band gap estimates from photoluminescence spectra of pod husk and N719 powders.

The UV-vis light absorbance performance of the dyes was studied by measuring the absorbance of bare/pristine TiO_2_ P25 photoanodes and their corresponding absorbances after soaking them in the various dye extracts. The difference in absorbance between the pristine and dyed photoanodes was taken as the absorbance of the pristine dyes that adsorbed onto the photoanodes. The results are shown in Fig. 5a. Extracts from water, ethanol and acetonitrile/*t*-butyl alcohol showed broadband absorbance across the UV-visible region, while that from acetone to hexane absorbed various sections within the spectrum, and mainly within 400–500 nm region. This intense broadband light absorbance of water, ethanol and acetonitrile/*t*-butanol extracts gives these natural dyes their uniqueness (and comparable to N719 dye), in comparison to most other reported natural dyes^26–28^ that absorb only various sections of the solar radiation as exhibited by the extracts of acetone to hexane.

Their absorbances span across at least ∼400 nm-range within the solar spectrum, compared to ∼200 nm-range sections, for most reported natural dyes;^26–28^ which indicates the high potential of these extracts as good dye materials for high performing DSSCs. The slightly polar to non-polar solvent extracts of chloroform, toluene and hexane absorbed slightly in the visible region, but mainly in the UV region, which is typical of most natural dyes. These absorbance characteristics of the dye extracts are good indicators of dyes that can absorb a great part of the solar radiation (with the characteristic effective range of 350–1000 nm)^15^ to generate many electron–hole pairs for enhanced current generation, ultimately giving highly enhanced PCEs in DSSCs.

The absorbance of the ethanol extract is of significant interest because it shows a very close overall light absorbance pattern to that of the high performing standard N719 dye. The TiO_2_ P25 spectrum was added for comparison, to show the independent absorbances of the pristine dyes. It absorbs in the UV region, which is typical of TiO_2_.^30,31^ However, it shows a broadband absorbance after 430 nm; this is attributed to the scattering effect of constructive interference effect of the incident radiation due to the different layers of the TiO_2_ nanoparticles across the thickness of the TiO_2_-layered photoanode.

### *I*–*V*

Table 1 shows the current–voltage (*I*–*V*) characteristics of the assembled DSSCs. The N719 dye DSSC showed a relatively high PCE of 6.22%. However, the natural dye extract DSSCs surprisingly showed relatively low PCEs of 0.07–0.19%. Although these results are comparable to many reported natural dye DSSCs of 0.05–4.2%,^21^ these are far lower PCEs than predicted, given their generally high and competitive light absorbances. However, the reported high performance natural dye DSSCs in literature use dyes of very high purity or dye combinations or modified dyes or a combination of these. An example are the various works of Wang *et al.*,^18^ where they used a modified pure chlorophyll (chlorophyll a derivative (PPB-a)) in combination with carotenoids (10–20%) of different number conjugated double bonds, serving as spacers to neutralize the dye radical cation and to block the reverse electron transfer recombination effects. The pristine PPB-a dye had effective absorbance mainly at 350–450 nm and then at 650–700 nm ranges within the solar radiation spectrum, while the carotenoid-PPB-a combinations mainly absorbed within the range of 350–530 nm of different intensities. Nevertheless, a +β-carotene carotenoid in combination with PPB-a produced a DSSCs PCE of 4.2%.^18^

**Table 1:** Extracted *I*–*V* characterization parameters of dye extracts and N719 DSSCs

Extract	*J*_sc_ (mA cm^−2^)	*V*_oc_ (V)	FF	PCE (%)	*R*_TS_ (Ω)	*R*_sh_ (Ω)
Water	0.071	0.506	0.527	0.07 ± 0.01	1311.3	18 564.7
Ethanol	0.105	0.502	0.546	0.10 ± 0.01	803.7	11 825.8
ACN/*t*-BuOH	0.094	0.518	0.554	0.10 ± 0.01	831.9	15 151.1
Acetone	0.101	0.527	0.566	0.11 ± 0.02	937.3	13 933.2
Chloroform	0.128	0.606	0.686	0.19 ± 0.01	523.0	49 378.7
Toluene	0.089	0.583	0.724	0.13 ± 0.01	629.0	108 249.6
Hexane	0.102	0.529	0.681	0.13 ± 0.01	652.4	67 044.6
N719	3.41	0.715	0.722	6.22 ± 0.01	31.4	65 936.6

Considering the key individual parameters contributing to the performance of a solar cell, it can be seen that, generally, these cells exhibit low short circuit current densities (*J*_sc_), low open circuit voltages (*V*_oc_) and very high total cell resistances (*R*_TS_). However, their fill factors (FFs) are relatively good, with a minimum of 52.7% of the water extract to a maximum of 72.4% of the chloroform extract, which is better than even that of the N719 sample of 72.2%. These FF values are also better than those of many natural dyes;^19^ the works of Wang *et al.*,^18^ with a PCE of 4.2% exhibited a FF of 58%, and the ethanol extract of neem leaves by Sahare *et al.*^36^ gave a FF of 35% with a PCE of 2.83%. The FF (= the ratio of the maximum power from the solar cell to the product of *V*_oc_ and *I*_sc_) is a measure of the ability of the solar cell to deliver current and potential at the same time.^19^ Thus, it is not directly impacted by the effects of charge generation, transport and dynamics before power generation, and hence, its relatively good values. In other words, FF is affected only after photogenerated electrons are injected into the CB of the semiconductor material (TiO_2_ P25 in this case), such that, if these electrons recombine from the CB, or during transport from the CB to the load/electrical generation point, then a lowering of/poor FF is observed. Or, if after the successful injection of photogenerated electrons into the CB (which can produce high *V*_oc_ by moving the Fermi level upward with a resultant increase in the Fermi level-electrolyte reduction potential gap = *V*_oc_) and these injected electrons recombine before reaching the load/power generation point, then a poor/lower *V*_oc_ value is observed. Thus, these good FF values with low *J*_sc_ and low *V*_oc_ values, only indicate that most of the photogenerated electrons from these excellent light absorbing dyes never reached the CB of the TiO_2_, but with the few that were successfully injected, being efficiently transported (high *R*_sh_; no power leakage) to the load/power generation point with consequent good FF values.

The most significant of the low performance key parameters of these natural dyes is their very high *R*_TS_; with the best performing/lowest value being that of the chloroform extract, being, at least, 16× that of the N719 dye, and curiously, it gave the best PCE of 0.19%. The *R*_TS_ is very critical because it has a severe impact on the other key parameters as observed and explained as follows. The total cell resistance, *R*_TS_, is the total resistance of the cell from individual cell components and component interfaces; such as the bulk resistance within the semiconductor material (TiO_2_) coating, the resistance of the FTO substrates, the resistance of the wire conductors, charge transfer resistance at the dye/TiO_2_ interface, *etc.* A high *R*_TS_ can mean inability/inefficient injection of photogenerated electrons into the conduction band of TiO_2_ by the dye, or a lack of efficient electron transport through the TiO_2_ photoanode, a consumption of photogenerated charges by the dye (recombination), *etc.* or a combination of these, dissipating power; all of which will ultimately result in a low *J*_sc_, a critical value for high PCE.

The *V*_oc_ can be described as the difference between the reduction potential of the electrolyte and the Fermi level of the TiO_2_ (which is affected by the dye by the amount photogenerated electrons injected into its CB, with more electrons meaning the higher the Fermi level, and thus, increased *V*_oc_). The *V*_oc_ can also be described as the highest voltage a cell can produce under illumination when no current is drawn from it. Although the *V*_oc_s of the natural dyes are lower than that of the N719 dye (0.715 V), they are actually good in the niche of natural dyes. Out of 75 tabulated reviewed natural dye DSSCs in the work of Kumara *et al.*,^19^ only 7 (9%) had *V*_oc_s around 0.60 V, the rest (93%) exhibited *V*_oc_ values between 0.23–0.55 V. Thus, the *V*_oc_ values range of 0.50–0.61 V are actually high performing natural dyes in the *V*_oc_ parameter. Their lower *V*_oc_ values but comparable light absorbance to N719 dye may suggest inefficient injection of photogenerated electrons into the CB of the TiO_2_, which will reduce *J*_sc_ and increase resistance in the diffusion-conduction of electrons in the photoanode. In summary, the PCE is given by:

1 PCE = *J*_sc_ × FF × *V*_oc_/*P*_in_

where *P*_in_ = incident power.

Thus, this equation summarizes how the various key parameters ultimately contributed to the low PCEs of the natural dye extracts, the main parameter of interest.

The shunt resistance, *R*_sh_, is an indicator of current leakage (alternate current path for the photo-generated current) from presence of impurities in the cell or defects in the cell during fabrication. The 10 s megaohm (10 MΩ) range of values of both the natural dyes and N719 dyes cells indicate well-fabricated cells with little or no impurities (that consume photocurrent) in the cells.

The analyses so far indicate that the lower-than-expected PCEs of the natural dye are due to high total series resistance from cell components, with possible contributory effects from inefficient injection of photogenerated electrons into the CB of TiO_2_ and possible increased resistance in the photoanode due to fewer electrons in there for effective diffusion-conduction of the photogenerated electrons that would have been injected into the CB of TiO_2_.

### IPCE

To further investigate the observations made above, the incident-photon to power conversion efficiency (IPCE) on the DSSCs was carried out to observe which regions of absorbed solar radiation was, or was not, generating power. The results are shown in Fig. 5c, which seem to correlate the PCE results obtained, but still different from the light absorbance characteristics of the natural dye extracts.

The N719 dye shows power generation from ∼750 nm (corresponding to its light absorbance onset) to the UV region. However, the extracted natural dyes DSSCs generated most of their power in the limited UV solar radiance region, with a small amount generated between 350–550 nm of the visible region.

The above results seem to suggest that the natural dye extracts absorbed light only in the UV region. To verify this the bandgaps of the dyes were estimated *via* Tauc plots using diffuse reflectance spectroscopy light absorbance data of the pristine dyes from the dyed photoanodes of Fig. 5a. The results are shown in Fig. 5b. We assumed direct bandgaps for them because their direct band gap transitions calculations curves exhibited more linear edges than their indirect transition curves; the band gap of N719 is known to be direct^37^ and TiO_2_ P25 is both direct (rutile) and indirect (anatase).^31,38,39^ The band gap of TiO_2_ P25 obtained was 3.15 eV as similarly obtained by Coutts *et al.*,^31^ Lopez *et al.*^38^ and Fidalgo *et al.*;^39^ whiles that for N719 was 1.75 eV, similarly obtained by Angelis *et al.*^37^ of 1.58–1.91 eV (of about 18 different transitions) *via* a combination of modified DFT calculations and experimental studies, and Prima *et al.*^40^ of 1.63 eV from Tauc plot and 1.85 eV from electrochemical technique. These good estimates of the band gaps of TiO_2_ P25 and N719 served as a good baseline and good confidence in our approach to estimating those of the natural dye extracts. The bandgaps of water, ethanol, acetonitrile/*t*-butanol and acetone extracts were determined to be 1.82, 1.83, 1.88 and 2.25 eV, respectively, which are comparable to that of the N719 dye of 1.75 eV and reflect their UV-vis absorbance spectra. The less polar to non-polar solvent dye extracts had band gaps in the range of 2.80–2.86 eV, which corroborate their light absorbance and IPCE performances. These results suggest that the more polar solvent dye extracts have their absorbance onsets close to the visible region edge at the infrared side, and hence, should have generated power from there and across the visible-UV region range, contrary to what was observed in the IPCE results.

### PL

The photoluminescence (PL) spectroscopy (a direct band gap transition approach) of the pod husk and N719 powders were also measured to estimate the band gaps (using the Planck–Einstein relation of *hv* = 1240/*λ*) of the maximum peak wavelength as given in the inset figure of Fig. 5d. The PL bandgap estimation for the N719 dye is 1.56 eV, like that reported by Nazeeruddin *et al.* (1.55 eV),^34^ who first reported on this dye. The PL value of 1.56 eV has a closer reflection to the ∼790 nm wavelength onset light absorbance of N719 dye (Fig. 5a and ref. 37), and a Kubelka–Munk estimation from the UV-vis spectrum gives a value of 1.50 eV. The PL-estimated bandgap of the pod husk exhibited a broad range spectrum bandgap with about four distinguishable peaks and a median value of 2.07 eV. This reflects and corroborates the 1.82–2.86 eV range of band gap values obtained from the individual solvent dye extracts *via* Tauc plot in Fig. 5b.

With the obtained dye band gaps supporting the broadband light absorbance of many of these natural dyes, but limited UV region IPCE performance, the PL spectroscopy of the dyed photoanodes were then evaluated to know if these dyes generated photoelectrons, and if they did, then what were the dynamics of these photogenerated electrons. The basic principle of PL spectroscopy is that a light absorbing material is probed with the appropriate wavelength of light, that makes the material generate excited electron–hole pairs, and if these excited/photogenerated electrons are not transferred/captured, they return to their ground states in electron–hole recombination, releasing the exciting energy they absorbed. This released energy is what is detected as PL intensity. Thus, a high PL intensity means a high electron–hole recombination. The PL spectra of the photoanodes in Fig. 5d shows that the N719 dye has the least intense PL (lowest electron recombination). The work of Angelis *et al.*^37^ indicates that the dye π* orbitals of N719 dye form the LUMO of the dye and these are strongly overlap/couple with unoccupied states of TiO_2_ that form the CB of TiO_2_. This leads to an almost instant (femtosecond time scale^12^) injection of photogenerated dyes from the LUMO of the dye to TiO_2_. Thus, the low PL intensity of the N719 indicates that most of these photogenerated electrons were transferred to the TiO_2_ CB without recombining to elicit PL radiation. The next low intensity PL is pristine TiO_2_ photoanode that also spans into the visible region; these are mainly transitions from surface trap states within the band gap of TiO_2_. This actually contributes to the relative conductivity of TiO_2_.

Halidun *et al.*,^41^ have reported on the effect of extraction solvent modifying the band gap of natural dye. However, in this our work we think the different band gaps from the different solvent extracts is from different dye components in the pod husk powder being extracted into the different solvents based on the fundamental/basic science of extraction/partitioning of molecular compounds into solvents of extraction based on factors such as, polarity matching between the molecule and the solvent; solubility and partitioning of the molecule into the solvent; effect of pH and ionization to modify the solubility of the molecule into the extracting solvent; molecule-extraction solvent interactions such as hydrogen bonding, van der Waals forces and dipole interactions; *etc.* And this view is supported by the observance of different PL peaks correlating to different band gaps of different dye components in the broadband PL results of the pristine non-solvated pod husk powder in the inset spectra of Fig. 5d; and some nuanced differences in the FT-IR results of Fig. 7a, among the different solvent-extract dyes, and between them and the pristine pod husk powder. Nevertheless, we do not completely deny the possibility of modification of functional groups of dye components that can ultimately modify their optical properties such as their band gaps, and ultimately their performance, as we observed later, the modification of the performance of these dyes in Table 4(a) when their pHs were modified with ethylene diamine alkaline.

In Fig. 5d, all the PL spectra of the natural dye extracts have higher intensities than N719 dye spectrum, proving that there is a high charge recombination occurrence in these natural dyes than in the N719 dye, and thus, an indication of inefficient charge/electron injection into the CB of TiO_2_ by the natural dyes. However, this recombination will be different from the recombination that occurs after the successful injection of photogenerated charges into the CB of the semiconductor material component of the DSSC; which effect is usually observed in low FF, low *R*_rec_ and low electron lifetime (*τ*_rec_).

Thus, inefficient electron injection into the CB with consequent low cumulative electron charge/concentration, would mean high resistance to electron transport in the diffusion model of electron transfer in the photoanode, and low *J*_sc_. Thus, this corroborates the observed high series resistance and low *J*_sc_ values observed in the *I*–*V* characterization results of Table 1, and thus ultimately the low PCEs and IPCE performance. This is also evidenced in the fact that the best performing chloroform extract dye cell exhibiting the best (lowest PL intensity) among the natural dyes, and an almost perfect order of increasing PL intensity with decreasing PCE values. The relative IPCE in the UV region may be attributed to the relatively higher energy of these electrons transition in that region. Thus, this shows that, these natural dyes efficiently absorbed light, generated excellent amount of photoelectrons, however, they suffered poor electron transfer to the CB of TiO_2_. And this could be due to a poor band alignment between the LUMO of the dyes and the CB of TiO_2_, or from parasitic charge consumers in the dye or within the structure of the dye.

### EIS

**Fig. 6:**
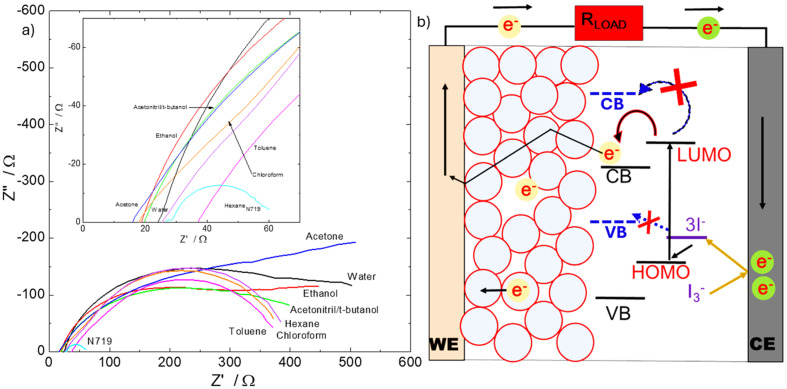
(a) Nyquist plots of electrochemical impedance spectroscopy measurements of DSSCs of the extracted natural and N719 dyes, inset: expanded left corner; (b) a schematic of the working principle of a DSSC showing inefficient or forbidden electron charge transfer (dashed blue lines and arrows) from possible inappropriate energy band alignments of the energy edges, LUMO and HOMO, of the natural dyes with the band edges of the TiO_2_ and reduction potential of the I^−^/I_3_^−^ electrolyte, respectively; compared to the normal electron transitions (solid lines and arrows) in a DSSC cell.

Based on the outcome of the PL studies we further studied the electrochemical impedance spectroscopy (EIS) of the DSSCs to try to identify which specific cell component(s) or interface(s) of the DSSCs is/are responsible for the high total resistance of the cells obtained in the *I*–*V* results. The results are shown in Fig. 6a and Table 2.

**Table 2:** Extracted parameters from electrochemical impedance spectroscopy measurements of DSSCs of the natural and N719 dyes^a^

Extract	*R*_S_ (Ω)	*R*_CE_ (Ω)	*R*_rec_ (Ω)	*R*_d_ (Ω)	*τ*_CE_ (ms)	*τ*_rec_ (ms)	*τ*_d_ (ms)	*η*_CC_ (%)
Water	24	0.7	350	127	0.80	8	50	93
Ethanol	19	0.8	366	52	0.80	10	126	95
ACN/*t*-BuOH	21	1.5	297	80	1.26	8	50	93
Acetone	16	2.0	320	102	0.80	6	79	93
Chloroform	18	0.7	330	23	0.63	20	100	95
Toluene	37	0.8	306	27	0.63	13	79	89
Hexane	26	1.4	324	33	0.80	20	79	92
N719	27	0.6	29	2	2.52	10	101	52

a *R*_S_ – series resistance due to the FTO substrates; *R*_CE_ – charge transfer resistance at the counter electrode/electrolyte interface for reduction of I_3_^−^ to 3I^−^; *R*_rec_ – charge resistance against recombination at TiO_2_/electrolyte interface; *R*_d_ – resistance to the diffusing redox species; *τ*_CE_ – electron lifetime at the CE; *τ*_rec_ – electron lifetime in the TiO_2_ photoanode; *τ*_d_ – ionic lifetime of diffusing redox species; *η*_CC_ – charge collection efficiency from conduction band to external circuit.

The Nyquist plots of all the samples start and intercept the real-axis, *Z*′, around the average value of 23.5 ± 6.27. Ω (Fig. 6a and Table 2). This is in the high frequency region (≳100 MHz) and hence, attributed to the ohmic resistance of the cell, *R*_s_, contributed by FTO substrates and the external circuit wires, which are both contributory resistances to the total series resistance (*R*_TS_) observed from the *I*–*V* characterization in Table 1. These values match those of pristine FTO substrates measured by EIS technique under the same conditions as the DSSCs.

Ideally, the Nyquist plot of a DSSC cell should consist of approximately three semicircles: the left-side high frequency end, representing the impedance/resistive dynamics at the counter electrode, the middle mid-frequency region representing the resistive dynamics at the photoanode, and the right-end low frequency side semicircle representing the resistive/diffusion dynamics of the redox electrolyte species.^42,43^

An obvious and curious observation from the Nyquist plots is the relatively large semicircles of the natural dyes over the N719 dye. This is mainly due to the mid-frequency region (∼10 000–100 000 Hz) electron dynamics impedance, attributed to charge transfer resistance against recombination at the TiO_2_/dye interface, *R*_rec_. This is given by the diameter of the middle semicircle on the real *Z*′-axis, given in Table 2. This resistance prevents photogenerated electrons in the conduction band of TiO_2_ from recombining with the electrolyte. Thus, generally, the larger they are the better because it will mean a very large value means almost no recombination. However, a very large *R*_rec_ value can also mean that there were no enough electrons in the conduction band to cause recombination and does thus, a very large value to indicate that there was negligible recombination. This is supported by well-established reality of DSSCs that recombination increases exponentially with increasing concentration of electrons in the conduction band of the semiconductor material.^33–35^ A second supporting evidence is from our own measurements of the EIS of the DSSCs without illumination, and those of the works of Bisquert^42^ and Nissfolk;^43^ where, without illumination, there are low concentrations of electrons in the CB of TiO_2_, and as a result such DSSCs exhibited similar very high *R*_rec_ values around 3000 Ω or more, and similar Nyquist plot shapes with wide mid-frequency semicircle diameters.

From Table 2, the high performing N719 dye DSSC has a *R*_rec_ value of 29 Ω, whiles those of the natural dye extracts are within very high values range of 297–366 Ω; indicating, and supporting the theory of, inefficient charge injection (or low concentration) of photogenerated electrons into the CB of TiO_2_ by these dyes. This is also supported by the Butler–Volmer relationship,^44^ which shows that transport and recombination resistances follow the Fermi level (or the concentration of electrons in the film/CB).^45–48^

Also, the *R*_CE_, *τ*_CE_, *τ*_rec_, and *τ*_d_, of all the cells are within the standard values for DSSCs;^25,33,43^ indicating that the natural dyes had no effect on the charge transfer from the counter electrode to the electrolyte, and there were no recombination effects at the counter electrode/electrolyte interface, in the photoanode or in the electrolyte by virtue of the good/standard values of their various electron lifetimes (*τ*).

Continuing from Table 2, the charge collection efficiency from the CB to the external circuits (*η*_CC_) of the natural dye extracts are higher (in 90% range) than that of the N719 dye (52%), and strangely high in nominal terms/relative to reported literature values.

This is attributed to the relatively low concentration of electrons in the CB of TiO_2_ in the cells of the natural dyes due to either inefficient charge injection by the dyes, or the relatively low amount of generated charge from low light absorbance dyes with relatively wider band gaps. Thus, with low charge concentration in the CB, they are more efficiently extracted, compared to the N719 dye that showed efficient charge injection into the CB and hence abundant/high charge concentration which also causes increased charge recombination; a normal phenomenon in DSSCs.

Another irregularity in the results of Table 2 is the high resistance values to diffusion of redox species in the natural dyes cells (in the 10 s and 100 s Ω, compared to 2 Ω for the N719 dye cell). We attribute this to three possible causes: (1) because of the inefficient charge transfer to the CB by the natural dyes, the uninjected electrons recombine with the holes in the HOMO of the dye (not recombination with the electrolyte) the electrolyte species are supposed to neutralize/combine with, causing accumulation of unoxidized redox species (I^−^) around the holes area that results in resistance for more of these redox species to flow there;^43^ (2) it could be due to poor band alignment of the HOMO of the natural dyes to the reduction potential of the electrolyte (Fig. 6b; dashed blue arrow); (3) the redox species are degrading the dye species. This is referenced from the work of Kumara *et al.*,^49^ where they suggested that there is degradation of natural dyes by iodine (I_2_ or I_3_^−^) of the I^−^/I_3_^−^ wet electrolyte. So, they used solid-deposited *p*-CuI electrolyte in their work, which showed increased performance of their cells. Thus, our natural dyes could also be experiencing decomposition from the I^−^/I_3_^−^ wet electrolyte leading to their high undesirable *R*_d_ values.

### FT-IR

**Fig. 7:**
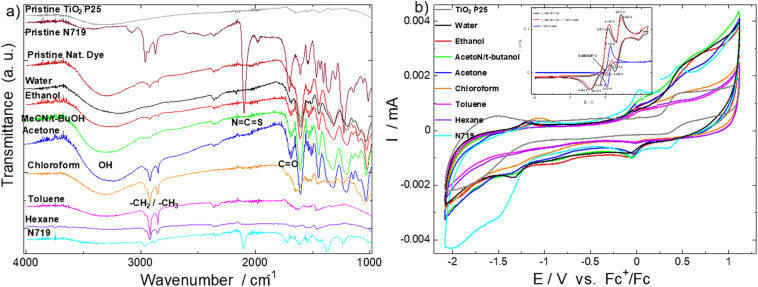
(a) FT-IR of the natural dyes, N719 dye and pristine TiO_2_ P25 nanoparticles from the photoanodes to show essential functional groups for DSSC application and nuanced differences among the natural dye extracts. (b) Cyclic voltammetry plots of the dyes scraped from the photoanodes and deposited on GC electrodes; inset: CV of pristine I^−^/I_3_^−^ electrolyte (black curve), ferrocene + electrolyte, and pristine ferrocene (blue curve) as reference.

To investigate the cause of the possible inefficient charge injection, we also carried out FT-IR characterization of the dyes on the dyed photoanodes to ascertain if they bear functional groups for anchorage and coupling to the TiO_2_ semiconductor. The results are given in Fig. 7a.

The pristine N719 dye powder spectrum had a broad peak between 3300 and 3700 cm^−1^, attributed to –OH group of its carboxylic acid; a sharp peak at 2100 cm^−1^, attributed to its thiocyanate, N=C=S, group stretching; and another sharp peak around 1700 cm^−1^, representing a stretching carbonyl, C=O, group (from its carboxylic ligand) (Merck: IR Spectrum Table & Chart). These are all anchorage ligands and are also observed in the spectrum of the N719 dye sample scraped from its dyed photoanode, but with reduced intensities attributed to most of these functional groups having been used to anchor to the TiO_2_. The spectra of the pod husk powder and extracts show broad band OH stretching peaks around 3320 cm^−1^ and sharp C=O stretching peaks around 1690 cm^−1^. These observations suggest the dye extracts have anchoring ligands of, at least, OH and C=O groups, and are most likely those of organic acids, which gives their extracts acidic pH range of −2.00 to 6.60.

The FT-IR results of Fig. 7a shows stretching peaks of –CH_2_, and –CH_3_ around 2912 and 2840 cm^−1^, respectively,^26^ for all the samples, indicating their organic nature. The stretching peaks of –CH_2_ and –CH_3_ for the N719 dye come from its tetra butyl ammonium (TBA) moiety. These results agree with the phytochemical-biased results of the works of Abagale S. A. *et al.*^22^ and Fayinminnu O. O. *et al.*,^23^ which identified families of compounds such as flavonoids, saponins and tannins as the main components of the polar extracts, with suspected presence of low molecular weight carboxylic acids, alkenes, alkynes, alcohols, ketones, aldehydes, nitro compounds, esters, ethers, amides, *etc.* However, we believe there are other compounds of better light absorbing characteristics than these identified families of compounds, or at least a combination of them, since this work's extracts (especially the polar solvents extracts) show excellent light absorbances than similar family of compounds reported in the review work of Kumara *et al.*^19^ Thus, the natural dyes have reasonable functional groups for anchorage to the TiO_2_ semiconductor component for charge transfer. However, these results cannot determine how appropriate the LUMOs of these natural dyes align and couple with the CB of TiO_2_; these will require the identification of the active dyes in these extracts, which is beyond the scope of this work.

### Photoanode thickness

Another parameter we thought could affect the performance of these natural dye extracts was the thickness of the photoanode, with the hypothesis that these natural dyes could modify the electron dynamics to cause their cells to have shorter electron diffusion lengths, thus causing the loss of electrons before they are fully extracted. Thus, we reduced the thickness of the photoanodes from 8 μm to 5 μm, giving the results in Table 3. These results show a general increase in the PCEs of the natural dyes, with accompanying reduction in total series resistance (*R*_TS_) (some as much as 40% reduction), whiles that of the standard reference N719 dye sample showed a significantly reduced PCE and increase in *R*_TS_. These interesting observations are significant we observed some improvements in their other parameters of *J*_sc_, *V*_oc_, and FF. We believe these parameters improvements in the natural dyes are mainly due to increased electrons in the photoanode for the natural dyes, and reduction in same parameters in the N719 dye due to reduction in photoelectrons in the photoanodes, as already explained under the EIS section above. A reduction in the thickness of the photoanode means a reduction in the total amount/volume of dye and ultimately the total volume of photogenerated electrons per photoanode material unit. Thus, this will lead to some increase in resistance in the photoanode, observed in the increase in *R*_TS_ value from 31 to 57 Ω in the N719 (∼84%), reduce FF from 0.72 to 0.66 V, and reduce *J*_sc_ from 3.4 to 2.5 mA cm^−2^, ultimately resulting in a reduced PCE of 4.2% from 6.2%. This shows how critical the concentration of electrons in the photoanode/TiO_2_ CB for efficient performance of the DSSC, supported by the presence of this parameter in the Butler–Volmer relation.

**Table 3:** Extracted parameters from *I*–*V* characterization of 5 μm thick photoanodes

Extract	*J*_sc_ (mA cm^−2^)	*V*_oc_ (V)	FF	*R*_TS_ (Ω)	PCE (%) (5 μm)	PCE (%) (8 μm)
Water	0.086	0.472	0.633	769	0.09 ± 0.01	0.07 ± 0.01
Ethanol	0.145	0.489	0.538	733	0.13 ± 0.01	0.10 ± 0.01
ACN/*t*-BuOH	0.144	0.498	0.601	551	0.15 ± 0.01	0.10 ± 0.01
Acetone	0.182	0.467	0.411	868	0.12 ± 0.01	0.11 ± 0.02
Chloroform	0.135	0.543	0.715	538	0.19 ± 0.01	0.19 ± 0.01
Toluene	0.168	0.556	0.736	387	0.24 ± 0.02	0.13 ± 0.01
Hexane	0.110	0.548	0.733	598	0.15 ± 0.01	0.13 ± 0.01
N719	2.493	0.718	0.661	57	4.20 ± 0.05	6.22 ± 0.01

In the case of the natural dyes the reduction in the thickness/photoanode material seemed to have increased the amount of electrons per unit volume of photoanode material, even with inefficient charge injection, thus improving the conductivity of the photoanode, resulting a general improvement in all the other key parameters of *J*_sc_, *V*_oc_ and FF, that ultimately results in increased PCEs at various levels in the natural dye DSSCs. This clearly indicates how crucial it is for there to be electron charge abundance, or at least some threshold amount, of electrons in the conduction band of TiO_2_/photoanode material for an optimal performance of all the other key cell parameters of a DSSC cell. Thus, a cell, or dye for that matter, may generate abundant amounts of electrons, however, if they cannot be transferred into the CB of the semiconductor material, the power conversion efficiency of the cell will be low and even worsened by its adverse consequent cascading effects on other key parameters of the cell. These observations are supported by the relation of eqn (2); fewer cumulative electron charge number in the conduction band will result in increased resistance, since film conductivity (*σ*) is directly proportional to cumulative electron charge in a conduction band (*n*_CB_) and mean free path.^14,50^

2 *σ* = *n*_CB_*eμ*

where, *e* is elementary charge (cumulative electron charge) and *μ* is electron mobility.

This effect is further heightened in the DSSC as a solar cell because of two major dynamics in the DSSC: (1) charge conduction is achieved *via* diffusion (a charge concentration effect), and (2) charge separation in the DSSC; charge separation is not achieved by a p–n junction barrier, but by the relative speeds of electron charge transfers at the various interfaces in the cell. Thus, fewer CB electrons leads to reduced conductivity with consequent high resistance, leading to poor charge separation and increased chance of various types of charge recombination. Thus, the improved performance in the thinner 5 μm-photoanode DSSCs of the natural dyes indicates there was improvement in electron kinetics attributed to improved available/abundance of photoelectrons. The improvement could have been also attributed to dye-aggregation; however, we studied this (which results are not added here for the simplification of the manuscript) *via* dye soaking for 12 h, 24 h and 48 h, and there were no significant differences among the *I*–*V* characterization of the DSSCs of these samples.

### pH modification

Another attempt to find the possible cause for the relatively low performance of these natural dye cells was the effect of pH on the dye extracts and the performance of their cells. The pH of the dyes is known to affect the performance of DSSCs^51–53^ by various effects such as protonation or deprotonation of the dye that may affect its anchorage, coupling with the semiconductor component, and even the HOMO, LUMO and band gap energetics. Thus, we modified the pH of our dye extracts from the original acidic states to various basic states using an organic base, ethylene diamine. The spiking with base was gradual, to allow for equilibration, over two days. The pHs were monitored using an organic based probe (Horiba Scientific Laqua) for the organic-solvent-based extracts. These modified dyes were then used to dye 5 μm-thick photoanodes and assembled into DSSCs. The results are shown in Table 4(a), compared to Table 4(b) (from Table 3 with a column on pH).

**Table 4:** *I*–*V* results of (a) basic dye extracts, compared to (b) their acidic counterparts

Extract	pH	*J*_sc_/(mA cm^−2^)	*V*_oc_ (V)	FF	*R*_dc_ (Ω)	*η* (%)
**(a)**						
Water	7.05	0.104	0.515	0.741	553	0.14 ± 0.01
Ethanol	7.50	0.119	0.503	0.610	656	0.13 ± 0.01
ACN/*t*-BuOH	7.53	0.134	0.517	0.692	508	0.17 ± 0.01
Acetone	7.91	0.186	0.529	0.315	1786	0.11 ± 0.01
Chloroform	8.02	0.131	0.568	0.705	489	0.19 ± 0.01
Toluene	7.01	0.115	0.536	0.729	540	0.16 ± 0.02
Hexane	7.33	0.128	0.558	0.728	489	0.18 ± 0.01
N719	7.62	0.382	0.606	0.719	197	0.59 ± 0.01

**(b)**						
Water	6.25	0.086	0.472	0.633	769	0.09 ± 0.01
Ethanol	4.88	0.145	0.489	0.538	733	0.13 ± 0.01
ANC/*t*-BuOH	5.50	0.144	0.498	0.601	551	0.15 ± 0.01
Acetone	5.68	0.182	0.467	0.411	868	0.12 ± 0.01
Chloroform	3.70	0.135	0.543	0.715	538	0.19 ± 0.01
Toluene	−2.00	0.168	0.556	0.736	387	0.24 ± 0.02
Hexane	−1.80	0.110	0.548	0.733	598	0.15 ± 0.01
N719	6.60	2.493	0.718	0.661	57	4.20 ± 0.05

The results show mixed effects based on the extract, with some showing positive results, such as the water extract improving over 55% in PCE, from improvement in the key parameters; others negative, such as the N719 which shows a decline of about 86% in PCE, with degradation in the key parameters except *V*_oc_ (an indication modification of Fermi level of TiO_2_ from photoelectron generation).

Other samples such as the acetone and chloroform extract did not exhibit any significant change in PCE, although there were some significant changes in other key *I*–*V* parameters. The detailed effect of the pH modification on the various key parameters by the various dyes is beyond the scope of this work, however, the key information from these results indicate that pH modification of the dye extracts is another condition that can be optimized to obtain the best performance from these dyes.

### CV

We also carried out cyclic voltammetry to evaluate the feasibility of the reversibility of the oxidation-reduction (dye re-generation ability) of these dyes as required by a feasible light absorber material for solar cell application. The results are given in Fig. 7b.

We observed at least complementary oxidation-reduction peaks between the voltage range of −0.25 to 0.25 V (although some samples also show another complementary oxidation–reduction peaks between −1.5 to −1.0 V), with oxidation-reduction peak current ratios of almost 1. They also have oxidation–reduction peak separation voltages not wider than the ideal 57–59 mV for single electrochemically reversible redox couple compounds/molecules (or 70–100 mV range from high electrolyte resistance or quasi-reversible redox materials). These are the conditions for reversible or quasi-reversible redox couples^54^ and the dyes meet these conditions with peak-to-peak separation values of 34, 49, 62, 26, 42, 45, 45, and 80 mV for the dyes from water, ethanol, acetonitrile/t-butanol, acetone, chloroform, toluene, hexane and N719, respectively. Nazeeruddin *et al.*^51^ had 100 mV peak-to-peak separation in their work under similar conditions. The lower than the ideal 57 mV peak-to-peak separation values of the natural dyes are attributed to possible multiple reversible redox processes, with a second electrochemical step being thermodynamically more favorable than the first;^54,55^ and we think, for our work this is either due to an intrinsic nature of the dyes and/or probably the case due to the effect of the TiO_2_ component^56^ scraped along with the dye from the photoanode for these evaluations, such that there is transfer of the ejected electron from the dye to the TiO_2_ within the electrochemical cycle. Thus, these results indicate that the active components of the natural dye extracts have the capacity to oxidize and re-generate (be reduced) under light illumination in a cyclic fashion to function as light absorbing materials for application in solar cells. These voltammograms align with similar works done by Al-Eid, *et al.*,^57^ Wanwong *et al.*,^58^ Pordel *et al.*,^59^ Fetouh *et al.*,^60^ and Jalali *et al.*^61^

### Band alignment

**Fig. 8:**
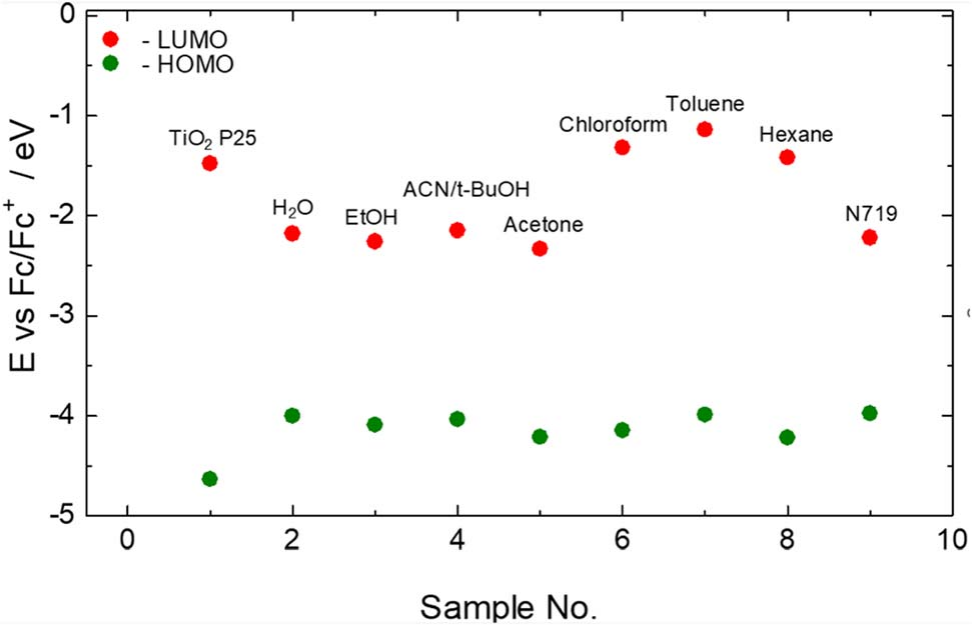
Energy level plots of dyes in comparison to that of TiO_2_ P25 to evaluate feasibility of charge injection into CB of TiO_2_ P25.

Another parameter we evaluated is the electrochemical oxidation onsets (*E*_ox_) of the dyes to estimate their band gap edges to be able to investigate their alignments with that of the CB of the TiO_2_ P25 for charge injection. The results are shown in Table 5 and Fig. 8. The HOMO and LUMO energy level edges were evaluated from eqn (3) and (4):^62–64^

3 HOMO = −(*E*_ox_ + 4.40) eV

4 LUMO = HOMO + band gap

**Table 5:** Electrochemical parameters obtained from voltammograms and optical band gaps of the dyes

Sample no.	Material	Band gap (eV)	*E*_ox_ (V)	HOMO (eV)	LUMO (eV)
1	TiO_2_	3.15	0.23365	−4.634	−1.48
2	Water	1.82	−0.39943	−4.000	−2.18
3	Ethanol	1.83	−0.30946	−4.090	−2.26
4	ACN/*t*-BuOH	1.88	−0.36541	−4.034	−2.15
5	Acetone	2.25	−0.18838	−4.211	−2.33
6	Chloroform	2.82	−0.25449	−4.145	−1.32
7	Toluene	2.85	−0.41147	−3.988	−1.14
8	Hexane	2.80	−0.18144	−4.218	−1.42
9	N719	1.75	−0.42449	−3.975	−2.22

The *E*_ox_ values were obtained from the curves of Fig. 7b from the voltage values of the intersections of the extrapolated tangents of the curves in the oxidation onset region.

From Table 5, the onset oxidation voltages of the dyes from polar solvents extracts (water, ethanol, ANC/*t*-butanol) and toluene (−0.31–−0.40 V) are very close to that of the N719 dye (−0.41 V), suggesting the relative ease with which electrons can be ejected from their HOMO orbitals, and thus, possible electron generation of electrons for electrical energy generation.

Fig. 8 seeks to show the feasibility of interfacial electron transfer from the dyes to the TiO_2_. In a relatively crude model, there are three (3) types of photosensitized electron injection from the dye to the semiconductor component, with effects of thermodynamics and kinetics: (1) direct electron injection from the LUMO, (2) electron injection from the HOMO, and (3) the multiple step injection, first to the VB or surface trap states and finally into the CB of a semiconductor.^64^ The direct injection route proposes that electrons are injected from the dye into the CB of a semiconductor (TiO_2_) and thus, the LUMO of the dye should be more positive in energy than the CB the TiO_2_ for effective/efficient transfer. Ooyama *et al.*^65^ suggest that the appropriate difference between the LUMO level of the dye and the CB of TiO_2_ should be more than 0.2 eV. From Table 5 and Fig. 8 it's only the chloroform, toluene and hexane extracts that have their LUMO levels above the CB edge of TiO_2_ P25, and unsurprisingly, they are the samples with the highest PCEs. The LUMO-CB difference for these samples are 0.16, 0.34 and 0.06 eV, respectively, which correlate their increasing PCEs (0.19, 0.24 and 0.15%, respectively, from Tables 3 and 4(b)) with their increasing LUMO-CB difference, in relation to the proposed optimal LUMO-CB difference >0.2 eV by Ooyama *et al.*^65^ The rest of the natural dyes have their LUMO levels/edges below the CB edge of TiO_2_, nevertheless, they still exhibited some appreciable power conversion PCEs, especially the N719. This observation suggests that electron transfer from the LUMO of dyes to the CB of the semiconductor material still occurs even if the LUMO is below the CB, albeit inefficiently/ineffectively; but the closer or above the LUMO is to the CB the more effective the transfer. This is further corroborated by the fact that, among the natural dye extracts with their LUMOs below the CB of TiO_2_, the LUMO of the ACN/*t*-butanol is closest to the CB and thus has the highest PCE of 0.15% among them.

These observations may then require a call onto the second and third crude injection models: such that in the second type electrons will be injected directly from the HOMO of the dyes directly into the CB of the TiO_2_. However, per the energy levels in Fig. 8, this may not be likely in our work since all the HOMO levels of the dyes are relatively far from the CB of TiO_2_, because in this second type of injection, their requirement is that the HOMO levels would have to be more positive than the CB energy level. Thus, we are left with the third type where electrons can be injected into the VB of the TiO_2_ from the dye that makes such electrons more liable for excitation into the CB; or injected into the common surface trap states of the TiO_2_ due to oxygen vacancies,^33^ and although these are usually non mobile/nontransferable electrons, a few will eventually be transferred to the CB; or the limited UV radiation of the solar radiation actually exciting electrons directly from the VB to the CB; or any combination of these electron transfer routes. However, these routes are not the conventional electron injection routes by basic electron kinetics, thus will be very inefficient, as observed in the results of extracts with high light absorbance and photoelectron generations.

A peculiar case is the LUMO level of N719 dye (−2.22 eV *vs.* Fc^+^/Fc) that places it 0.74 eV lower than the CB of TiO_2_ P25. This is unexpected because it produced the best PCE of 4.20% (from the 5 μm-thick photoanodes batch), although representing a 32% reduction in PCE from an initial value of 6.22% (of the 8 μm batch); which was explained, previously, to cause some concentration effects. Many other works have reported results that show N719 dye LUMO higher than (of about 0.38 eV (ref. 66) magnitude), or mixed with, the CB of TiO_2_, including ref. 11, 37, 40 and 66. Many factors affect the electron configuration of DSSC components (such as the dye), electron transfer within dyes and from dyes to the semiconductor material, such as dye concentration, electrolyte medium, dye-semiconductor level of coupling, *etc.*;^37,50,67^ such that a lower concentration (nominal or total/volumetric) can lead to different/modified coupling and electron kinetics, that could modify the electron kinetics of the cell. Also, the N719 electron kinetics is quite complex such as a Ru-metal-to-ligand charge transfer and a ligand-to-ligand transfer, which may subsequently be transferred to the N- or S-moiety of the CNS functional group, or to the –COO functional group before finally being transferred to the TiO_2_ semiconductor,^37,50^ resulting in a number of energy bands and many light excitation transitions such as a range of 1.90 eV to 4.00 eV.^37,50^ Thus a 2.5 eV transition will result in an N719 LUMO at −1.47 eV, placing it above the CB of TiO_2_ (−1.48 eV) and thus, resulting in effective charge transfer. In addition, the quantum state of molecular systems/electron transitions are described by three wave functions, electronic, nuclear/vibrations and spin, such that even with a lower LUMO than the CB of TiO_2_, vibrational effects could actually stretch/vibrate the orbitals more than the 0.74 eV LUMO-CB deficit (from Table 5) to give effective charge transfer into the CB of TiO_2_. Thus, the emphasis here is that, as much as the results of the N719 dye is peculiar from Table 5 and Fig. 8 results, it is still a possibility with effective charge transfer. However, this may require more investigation into these various possibilities.

## Conclusion

This study investigated the potential of seven (7) natural dyes extracted from the pod husk of *Parkia biglobosa*, using seven different solvents; in addition to the standard N719 dye as a reference, for application in dye-sensitized solar cells (DSSCs). The results suggest the extracted dyes were of different compositions with a number of them exhibiting excellent broadband light absence. Their bandgaps range from 1.82 to 2.85 eV, compared to 1.75 eV of the synthetic N719 dye. The high intensity broadband light absorbance within the solar radiation spectrum (comparable to, or better than, that of the high performing N719 dye) sets these natural dyes (especially, the water, ethanol and ACN/*t*-BuOH extracts) as unique from other reported natural dyes which usually absorb a total of just 200 nm-range, or a series of short-range wavelengths, within the solar radiation spectrum; making them high potential dyes for DSSCs. The power conversion efficiencies (PCEs) of the natural dyes ranged from 0.07% to 0.19% relative to 6.22% of the N719 dye. A reduction of the photoanode-thickness from 8 μm to 5 μm enhanced PCEs by 28–84%, to PCEs range of 0.9–0.24%, compared to a reduced PCE of 4.20% for N719 dye (32% reduction). These results of the natural dyes align with, and at the same time, show better performance than typical natural dyes of PCE range of 0.05–4.2%; with these reported high performing natural dye DSSCs usually consisting of high purity dyes or composed of dye combinations.

Nevertheless, the values of these natural dyes in this work fall short of the anticipated high performance, given their excellent light absorbances and bandgaps. Extensive characterizations to ascertain the cause of the unexpected lower PCEs included, UV-vis/band gap evaluation, current–voltage (*I*–*V*) evaluation, photoanode thickness modification, incident-photon to power conversion efficiency (IPCE), photoluminescence (PL), electrochemical impedance spectroscopy (EIS), FT-IR, and cyclic voltammetry (CV) [to evaluate the onset oxidation and bands alignments relative to the CB of TiO_2_]. The conclusion from these evaluations indicate that the main causes of the lower cell performances of these natural dye extracts were extremely high series- and ionic diffusion-resistances, due to poor bands alignments of the natural dye extracts to those of the TiO_2_, and possibly, to the reduction potential of the I_3_^−^/I^−^ redox couple. There were also indications of dye degradation from the effects of the redox electrolyte.

These findings suggest that finding an appropriate semiconductor with a lower and appropriate CB band than TiO_2_, such as WO_3_, SnO_2_, ZnO_2_, Bi_2_WO_6_, *etc.*, and an alternative appropriate electrolyte with less corrosivity and appropriate band alignment with the HOMO of the dyes, such as solid-deposited *p*-CuI, Fe^3+^/Fe^2+^, Cu^2+^/Cu^+^, Co^3+^/Co^2+^, *etc.* there is a high possibility of obtaining excellent DSSC performance with high PCEs from these natural dyes, based on their excellent competitive light absorbance with the high performing N719 dye.

Thus, this work presents results on never-explored natural dye extracts that have demonstrated remarkable potential as sensitizers for DSSCs, exhibiting excellent light absorbance characteristics, impressive stability, and promising *I*–*V* parameters. However, their performance is hampered by poor band alignment to TiO_2_ semiconductor material used, and indicative degradative and poor band alignment to I_3_^−^/I^−^ electrolyte. These challenges caused inefficient injection of the abundant photogenerated electrons into the conduction band of TiO_2_, and impede dye regeneration, respectively. Addressing these challenges requires strategic optimization, including electrolyte composition and electrode modifications to enhance charge transfer dynamics, mitigating losses associated with poor charge injection efficiency and poor ionic species diffusion/dye regeneration.

Despite these hurdles, the continued exploration of natural dyes offers exciting opportunities for sustainable and eco-friendly energy solutions. The commercial viability of natural dye-based DSSCs is currently limited due to their lower efficiency compared to synthetic dyes, mostly from short range light absorbance within the solar spectrum; making the dyes in this work unique. However, natural dyes in general, offer critical and unique advantages that could make them attractive for niche applications and future advancements. These advantages include, eco-friendliness, sustainability, low-cost, readily available, potential for indoor and low-light conditions applications, *etc.* By refining their electronic properties and optimizing device architectures, with other proposed solutions such as co-sensitization, chemical modification and alternative appropriate electrolytes, the integration of natural dyes into DSSCs can pave the way for high-performance, cost-effective photovoltaic technologies. Regarding this work, future research should focus on overcoming charge injection limitations to unlock the full potential of these abundant and renewable sensitizers, isolate the pure components of these dyes to further understand their electron, optical and electrochemical dynamics, that will open more opportunities for modification and optimization of these dyes. Modifications that can include molecular engineering, co-sensitizers, and improved interfacial tuning between the dye and semiconductor, *etc.*, for them to achieve the predicted high-performance potential they possess. And it will be advisable to work on a single extract at a time, for more in-depth evaluations.

## Author contributions

Conceptualization: PN. Resources: AM, GK, KN, TWK, GH, NM, PKM and GN. Data curation: PN, AM, GK, KN and GH. Formal analysis: PN and GK. Investigation: PN. Writing – original draft: PN. Writing – review & editing: PN, AM, GK, TWK, NM, PKM and GN. Supervision: AM.

## Conflicts of interest

There are no conflicts of interest to declare.

## Data Availability

The data that support the findings of this study are available from the corresponding author upon reasonable request. Because these data are in many different files and different formats and are not on a public URL, in addition to privacy restriction, the data are not publicly available.
